# Illuminating the Petite Picture of T Cell Memory Responses to *Listeria monocytogenes*

**DOI:** 10.1155/2013/121684

**Published:** 2013-09-22

**Authors:** Saba Tufail, Khan Farheen Badrealam, Mohammad Owais, Swaleha Zubair

**Affiliations:** ^1^Interdisciplinary Biotechnology Unit, Aligarh Muslim University, Aligarh, Uttar Pradesh 202002, India; ^2^Women's College, Aligarh Muslim University, Aligarh, Uttar Pradesh 202002, India

## Abstract

The ease to culture, moderately less safety constraints in handling, and above all, hurdle free induction of an anticipated infection in mouse rendered *Listeria monocytogenes* the rank of a model organism for studying a variety of host immune responses. *Listeria monocytogenes* being an intracellular pathogen evokes potent CD8 T cell response during which CD8 T cells pass through a massive expansion phase. This is generally followed by contraction phase wherein majority of activated cells undergo apoptosis leaving behind a population of memory CD8 T cells that has potential to confer enhanced protection upon reencounter with the same pathogen. Functional attributes of various cytokines, transcription factors, receptors, adaptors, and effectors pertaining to the generation of robust memory T cell response have begun to be unravelled for better understanding of memory and opening avenues to create superior vaccine strategies. This review is an attempt to unveil related discoveries along with updating recent advances on this issue.

## 1. Introduction

*Listeria monocytogenes *is a Gram-positive facultative intracellular bacterium and the causative agent of listeriosis, the most pernicious amongst various food borne infections in humans accounting for about 20–30% deaths worldwide [1]. Listeriosis is characterized by systemic dissemination of the heavy ingested inoculum of bacteria from gut to systemic circulation to various organs including brain manifesting in the establishment of gastroenteritis, septicaemia, and meningoencephalitis. Moreover, *Listeria monocytogenes* has a profound predilection for disseminated infection during pregnancy [2–4] which is attributable to the induction of fetal-placental infection triggering stillbirth, spontaneous abortion, premature delivery, and neonatal infection [5].

*Listeria monocytogenes* disseminates infection by cell-to-cell mode of spread along with being endowed with the capability of infecting both nonprofessional phagocytes (gut epithelial cells) and professional phagocytes (macrophages) [6]. The notoriety of the pathogen is associated with its ability to make macrophage cytosol its replicative niche [7], and it does so by disrupting the internalization vacuole exploiting its pore forming toxin, listeriolysin O (LLO), curtailing the fusion of the vacuole to hydrolytic phagosome [1]. The cytosolic abode and cell-to-cell spread protect the pathogen from extracellular milieu and safeguard it against antibody onslaught and complement attack. The intracellular niche occupied by *Listeria monocytogenes* leads to induction of potent CD8 T cell response wherein CD8 T cells proliferate and differentiate to effector cells in order to contain the infection. CD8 T cells play centrestage in the control and obliteration of intracellular pathogens [8]. Moreover, dendritic cell cross-priming of CD8 T cells is of chief importance in alarming cellular immune responses to *Listeria monocytogenes *infection. Once the infection is eliminated, the CD8 T cell population begins to contract as the bulk of the *Listeria*-specific CD8 T cells undergo apoptotic cell death; therefore, a skimpy population (5–10%) of *Listeria-*specific CD8 T cells is left to enter the memory T cell pool, and it is this memory T cell pool that confers better protection upon rechallenge with *Listeria monocytogenes* [9]. The CD8 T cell memory pool generated so forth does not carry a homogenous population rather a heterogeneous one which can be separated into central memory T cells (T_CM_) carrying high proliferation capacity but reduced immediate effector function and effector memory T cells (T_EM_) carrying low proliferation capacity but profound immediate effector function [9–13]. The heterogeneous CD8 T cell memory population is based on the expression of CD62L and CCR7; T_CM_ is CD62L^high^ and CCR7^+^ while T_EM_ is CD62L^low^ and CCR7^−^ [14].

The focal point of this review is T cell memory response to *Listeria monocytogenes* infection, and the reason behind choosing *Listeria monocytogenes* as the centrestage organism for this review is its exploitation as a model organism to study immune response against intracellular pathogens, and this is owed to the genetic manipulability of this microorganism and availability of overwhelming information on its pathogenesis [6, 15]. Exploiting the mutability of *Listeria monocytogenes*, its avirulent strains have been created which however retain the capacity to induce potent and protective acquired immune response and help deciphering a wider scope of immune response to intracellular pathogens [6]. 

**Figure 1:**
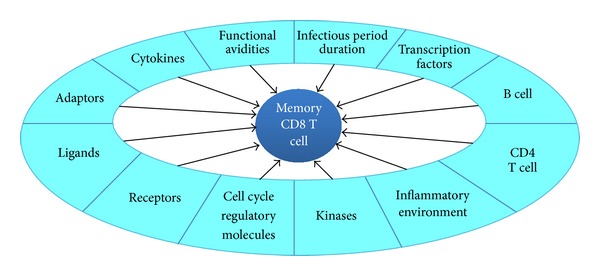
Illustration of various factors regulating the generation of memory CD8 T cells in response to *Listeria monocytogenes* infection as detailed in the text.

Herein, first we detail the generation of memory T cells in response to *Listeria monocytogenes *infection enumerating the “models” proposed for their differentiation. Second we review the role of various cytokines, transcription factors, receptors, adaptors, and effectors in the generation of a robust CD8 T cell memory response along with highlighting the recent breakthroughs. Further, we discuss the contribution of CD4 T cells and B cells in generation and maintenance of CD8 T cell memory response. Lastly, we enumerate how understanding memory response opens avenues for development of better vaccines.  Figure 1 gives an overview of the factors playing crucial roles in generation of memory CD8 T cells.

## 2. Generation of Functional Memory CD8 T Cells

### 2.1. Models of Memory CD8 T Cell Generation

It is very well documented that CD8 memory T cells play an indispensable role in controlling intracellular pathogens like viruses and certain bacteria including *Listeria monocytogenes*. As already discussed, postelimination of primary infection, the host harbours two distinct CD8 T cell memory subpopulations: T_EM_ and T_CM_. Two conflicting models for memory T cell differentiation have been proposed: one being “linear differentiation model” and the other being “progressive differentiation model.” According to linear differentiation model, memory T cells develop in continuum from naive cells which get activated upon antigen encounter and give rise to T_EM_ which finally progress to T_CM_. T_CM_ represents the memory T cell subpopulation that confers enhanced protection upon reencountering the same pathogen [16, 17]. The progressive model describes that T_CM_ generation bypasses the T_EM_ stage and arises directly from naive cells [16, 18]. According to this model, the “signal strength” to TCR (T cell receptor) during priming and expansion phase determines the fate. It is proposed for this model that, as a consequence of strong activating signal strength, T cells acquire full effector function and lose their potential to proliferate and survive. T_CM_ is generated in response to weak signals and provides precursors for rapid generation of antigen-specific effector cells, whereas T_EM_ has been proposed to develop as a consequence of intermediate signal strength. A study conducted by Teixeiro et al. [16] showed results falling in line with the progressive model. They have demonstrated that it is the differential TCR signaling in *Listeria monocytogenes *infection which determines CD8 T cell memory versus effector development. They exploited a mutant TCR transgenic model infected with *Listeria monocytogenes*and found that point mutations in the TCR *β* transmembrane domain although could not impact primary effector CD8 T cell response, they ensued in impaired development and function of CD8 memory T cells.

Different experimental systems and methods used to define phenotype and purify T cell subsets have been attributed for the existence of such contrasting views [19]. Problems with infection models have also been proposed to be the cause of deviating views, as in the case of chronic persistent viral infections; details about potential in vivo antigen (re) exposure are not clear, and frequent antigen encounters may increase the diversity of expression patterns for some markers [19]. Since linear model is often taken into account while describing antigen-specific T cell memory response, the details summarized herein are majorly in the light of this model.

### 2.2. Functional Avidities and Differentiation of Memory CD8 T Cells

High functional avidities determine the differentiation and longevity of memory T cells to accomplish the memory program. In an earlier study, *Listeria monocytogenes* specific effector and memory CD8 T cell populations have been investigated with respect to TCR repertoire [20]. The heterogenous CD8 T cell population (effector and memory CD8 T cells) comprises cells specific for immunodominant epitopes and expressing a broad spectrum of TCRs. Interestingly, TCR repertoire expressed during the primary response is retained by the memory T cells. However, during clonal expansion of memory T cells after reinfection with *Listeria monocytogenes*, the broad spectrum of TCR repertoire is narrowed rendering development of T cells carrying higher avidity for antigen. It is quite feasible that higher avidity memory T cells would have selective advantage on rechallenge, but the comprehension of precise mechanisms rendering alteration of TCR remains elusive. Furthermore, in a recent study, some researchers have also investigated whether CD8 T cells stimulated by low affinity ligands give rise to memory T cell population [21]. They chose a model in which TCR transgenic OT-1 cells were stimulated by five different altered peptide ligands (APLs) which were derivatives of original OT-1 ligand SIINFEKL (N4) and differed in the potency to stimulate OT-1 cells. Mice were infected with recombinant *Listeria monocytogenes* strains expressing chicken Ova protein containing APL (*Listeria monocytogenes*-APLOVA) in place of the N4 epitope. After 138 days of OT-1 cell stimulation by various APLs, a finite population was found to remain stable. Recombinant vesicular stomatitis virus expressing Ova (VSV-N4OVA) challenge rendered significant expansion of the OT-1 cells previously exposed to any of the APLs. On the contrary, far fewer OT-1 cells were observed after VSV-N4OVA challenge in mice that initially received a wild-type *Listeria* infection, which suggests that all the APLs generated memory cells. Transfection of N4 and V4 memory OT-1 cells into naive hosts led to comparable expansion of both after *Listeria monocytogenes*-N4OVA rechallenge. Interestingly, both memory populations also responded similarly to *Listeria monocytogenes*-V4OVA rechallenge. Thus, not the priming antigen rather the strength of the recall stimulus determines the expansion in the recall response. The findings enumerate that even very weak TCR-ligand interactions are enough to drive the formation of functional memory T cells and support the reports indicating that lymphopenia-driven homeostatic expansion, a manifestation where T cells also encounter only very weak TCR ligands, generates functional memory T cells [21].

### 2.3. Memory CD8 T Cell Differentiation Is Rapid and a Function of Duration of Infectious Period

It has been reported that *Listeria*-specific CD8 T cells are present during initial days after primary immune response, and their extensive potential to proliferate after antigen reencounter indicates that memory T cells undergo rapid differentiation [22, 23]. Moreover, CD127 (also known as the IL-7R) expressing T cell subsets seemingly appear at early time points in response to *Listeria monocytogenes* infection which further falls in line with the notion that memory T cell development is rapid [20, 24]. Williams and Bevanhave reported that duration of infectious period of *Listeria monocytogenes *dictates the programming of CD8 T cells to memory T cells [25]. Although, it is well known that brief exposure to antigen leads to expansion and differentiation of CD8 T cells, their work proves that full memory differentiation of CD8 T cells remains diminished when the infectious period is shortened. So, it appears that CD8 T cell memory programming is dependent on threshold infection duration. Furthermore, the finite size of memory CD8 T cells generated after *Listeria monocytogenes* infection has also been investigated because the number of memory cells formed is in direct corelation with the level of protection rendered in the infected host [17, 26–29]. Hence, seemingly a protective number of effector and memory CD8 T cells are indispensable to fight various infections and malignancies.

### 2.4. Cytokines and Transcription Factors Required to Generate Robust Memory CD8 T Cells

It has been spoken loud that CD8 T cell memory formation is critical for developing protective immunity against reinfection but the signals required to program activated CD8 T cells to develop into memory cells remain obscure. CD4 T cell assistance [30, 31] and IL-2 signals during priming have been reported to play some role in the establishment of memory population [32]. A recent study supports the critical function of IL-12 and type I Interferon (IFN) in inducing CD8 T cell memory development in response to *Listeria monocytogenes* infection [33]. Xiao et al. [33] report that, within three days of the initial phase of naive CD8 T cell expansion in response to Ag, memory development by IL-12 is completely programmed but this indoctrination does not lead to formation of memory cell population expressing killer cell lectin-like receptor G1 (KLRG1); rather the majority cells express CD62L^high^ phenotype which is an attribute of central memory cells. Hence, three major classes of signals contribute to T cell activation and their transit to memory cell development, signal 1 from antigen stimulation of TCR, signal 2 comes from costimulatory signalling through molecules like CD28, and the signal 3 is derived from the above-discussed cytokines namely, IL-12 and type I IFN. Differential expression of the T-box transcription factor, T-bet, has been revealed to partly mediate the effect of inflammatory cytokines like IL-12 on effector and memory CD8 T cells [34–36]. Moreover, downstream transcriptional programs of Wnt signalling have been reported to play as signal 4 in modulating T cell responses [36]. Wnt proteins (secreted, lipid modified glycoproteins) regulate various cellular activities owing to their potential to activate multiple signal transduction pathways. Zhao et al. demonstrate that TCF-1 (T cell factor 1) and LEF-1 (lymphoid enhancer-binding factor 1), the effector transcription factors of canonical Wnt pathway in a manner similar to IL-7R*α* and CD62L, exhibit dynamic expression changes during expansion of antigen-specific CD8 T cells in response to *Listeria monocytogenes* infection [36]. They found naive T cells to express plenty of TCF-1 and LEF-1 which were however downsized in number in effector T cells but found to be upregulated again in memory T cells. The study revealed that memory CD8 T cells transitioned to a large population of secondary effectors which rendered rapid clearance of bacteria after rechallenging immunized mice with virulent *Listeria monocytogenes*. The finding clearly suggests that activation of the canonical Wnt pathway leads to generation of memory CD8 T cell population during initial immunization, ensuing in ameliorated protective immunity upon further challenge with the same pathogen. 

TCF-1 and LEF-1 transcription factors exhibit overlapping roles in thymocyte maturation and have also been found to regulate memory CD8 T cell differentiation and persistence, but a wider spectrum of their functions remains yet to be unravelled. Zhou and Xue made an effort in this direction when they studied the effect of double deficiency of TCF-1 and LEF-1 on generation of memory precursors in effector CD8 T cell populations in response to *Listeria monocytogenes* infection [37]. The double deficit totally abrogated the generation of KLRG1^low^  IL-7R*α* ^high^ memory precursors and CD8 effectors lacking TCF-1 and LEF-1 although could still express IFN-*γ*, granzyme B, and perforin, but TNF-*α* production was found to be hampered in them. The double deficient antigen-specific CD8 T cells exhibited an effector phenotype in the memory phase, and, in secondary expansion upon reencounter, they were severely impaired. So, it can be concluded that TCF-1 and LEF-1 regulate generation of memory precursors and protective memory T cells in unison.

As already discussed, IL-12 and IFN-*γ* induce protective immunity against *Listeria monocytogenes* infection by regulating memory CD8 T cell development as well as modulating magnitude of short-lived effector cells generated. A group recently examined the role of a related cytokine, IL-23, in this regard and concluded that, in the absence of IL-12, it can act as its partial substitute for generation of protective immunity against *Listeria monocytogenes* challenge [38]. Moreover, Sandau et al. demonstrated that IL-15 plays a crucial role in dictating the composition as well as maintaining the CD8 memory pool; that is, it regulates both qualitative as well as quantitative features of CD8 memory T cell population [39]. The researchers observed that, after boosting, mice lacking IL-15 could not give rise to a subset of effector memory cells, including a population of IL-7R*α* low cells which however were found to be in higher proportion among secondary memory cells in normal mice. IL-15 deficiency also induced changes within the IL-7R*α* ^high^  CD62L^low^ subset of secondary memory CD8 T cells, which expressed high levels of CD27 but minimal granzyme B. Moreover, including these qualitative changes, deficiency of IL-15 led to reduced cycle and impairment of Bcl-2 expression by secondary memory CD8 T cells which is indicative of a definite role of IL-15 in basal proliferation and survival of the memory cell pool. Moreover, IL-7 has also been found to regulate the differentiation of CD8 memory T cells following the effector phase [39].

Whereas shaping CD8 T cell memory requires some cytokines, there are others which either function conversely or leave no effect. IL-10 has been well documented for inducing downregulation of T cell responses [40]. Moreover, Biswas et al. demonstrated that, in the absence of IL-10, primary and memory CD8 T cell responses against *Listeria monocytogenes *infection are enhanced [40]. Furthermore, a little later, Haring and Harty evaluated antigen-specific CD4 and CD8 responses in the absence of IL-18 or IL-18R*α* to determine the role of this cytokine in development and homeostasis of T cells. Whereas they observed a regulatory function of IL-18/IL-18R*α* in CD8 T cell contraction, their experiments revealed that neither IL-18 nor IL-18R*α* is required for the generation, contraction, or maintenance of memory CD4 and CD8 T cells in response to infection with an attenuated strain of *Listeria monocytogenes* [41].

### 2.5. Inflammatory Environment Contributes to Memory CD8 T Cell Differentiation

Pathogen induced inflammatory environment has also been observed to control effector and memory CD8 T cell differentiation [14]. According to the differential model, short-lived effector cell (SLEC: IL-7R*α* ^low^  KLRG1^high^) and memory precursor effector cell (MPEC: IL-7R*α* ^high^  KLRG1^low^) are formed from an early-effector cell (EEC: IL-7R*α* ^low^  KLRG1^low^). Obar et al. [14] demonstrated that the composition of the inflammatory environment induced by *Listeria monocytogenes* and vesicular stomatitis virus (VSV) infections affects effector CD8 T cell differentiation. They observed that inflammation in addition to altering SLEC/MPEC differentiation also limited the differentiation of CD62L^low^ T effector memory cells that searingly affected the functionality of the effector CD8 T cell population and composition of the MPEC population. Moreover, they found that SLEC/MPEC differentiation was altered in a memory cell intrinsic manner as a consequence of multiple encounters with the same antigen. It leads to the conclusion that effector and memory CD8 T cell differentiations are regulated by the type of priming pathogen and the number of the times the cell encounters the same pathogen. 

### 2.6. Adaptors, Ligands, and Receptors Required to Shape CD8 T Cell Memory

The mechanisms that direct the induction and maintenance of memory T cells are not clear yet. Gads, an adaptor protein in TCR signalling, plays an indispensable role in TCR mediated Ca^2+^ immobilization [42]. Effect of Gads deficiency on CD8 T cell mediated immunity has also been investigated in *Listeria monocytogenes* infection [42]. During the initial phase, Gads^+/+^ and Gads^−/−^ CD8 T cells expanded to an equivalent level, although the expression of CD69 and CD25 was reduced in the absence of Gads. Additionally, Gads was albeit required to sustain the proliferative phase of immune response five days after infection, it played no role in differentiation of naive CD8 T cells into memory cells. 

Another study conducted by Pearce et al. reveals that TRAF6, an adaptor protein in the TNF receptor (TNFR) and IL-1R/TLR superfamily, regulates development of CD8 T cell memory following *Listeria monocytogenes* infection by modulating fatty acid metabolism [43]. They found that mice with T cell specific deletion of TRAF6 could generate robust CD8 effector T cell responses which were however defective in their ability to transit to memory CD8 T cell population. Moreover, their study revealed that expression of genes regulating fatty acid metabolism is altered in TRAF6-deficient CD8 T cells. Falling in line, in the absence of growth factors, activated CD8 T cells lacking TRAF6 display defective AMPK activation and mitochondrial fatty acid oxidation (FAO). They found that administration of antidiabetic drug metformin replenished FAO and memory CD8 T cell production in TRAF6 deficient mice, and, interestingly, this treatment could also ameliorate memory CD8 T cell generation in wild-type mice. 

A G protein coupled neuropeptide receptor, VPAC1 (vasoactive intestinal peptide/pituitary adenylate cyclase activating polypeptide receptor 1) has also be investigated for its regulatory role on CD8 T cells in response to *Listeria monocytogenes* infection [44]. It has been demonstrated that CD8 T cells responding to *Listeria monocytogenes* have downregulated expression of VPAC1, so downregulation of VPAC1 expression exhibits inverse relationship with CD8 T cell proliferation. Interestingly, VPAC1 expression normalised to naive level in primary but remained low during secondary, memory generation. 

According to a report, Fas death pathway has some share in modulating the function of memory CD8 T cells [45]. While a similar increase or decrease of CD8 T cell response is observed against *Listeria monocytogenes *infection in wild-type (WT) and Fas ligand (FasL) mutant mice, FasL mutant mice had mainly T_EM_ population in the long term when compared to WT mice that carried majorly T_CM_ population. Downregulation of IFN-*γ*, poor homeostasis, and antigen-induced proliferation were observed for memory CD8 T cells in FasL mutant mice. Interestingly, faulty programming or defective FasL expression on CD8 T cells is not responsible for impaired CD8 T cell memory in FasL mutants, but the deranged cytokine environment in FasL mutant mice played the culprit. Although adoptively transferred WT memory CD8 T cells could confer protection against *Listeria monocytogenes *in either the WT or FasL mutant hosts, memory CD8 T cells in FasL mutants could not deliver protection even in WT hosts. Hence, subjects carrying mutation in Fas pathway have impaired memory CD8 T cell function which can render them susceptible to recurrent or latent infections. 

### 2.7. Cell Cycle Regulatory Molecules Modulate the Generation of CD8 T Cell Memory

In addition to the above-mentioned regulatory mechanisms, cell cycle regulatory molecules have also been reported to be key regulators of T cell response [46]. p27^Kip1^, a cyclin-dependent kinase inhibitor, has been found to be a critical regulator of the CD8 T cell homeostasis as well as response to an acute viral infection [35]. Most importantly, p27^Kip1^, in addition to inhibiting the programmed expansion of IL-2 producing memory precursors, had its impact on the magnitude and quality of CD8 T cell memory. Singh et al. [46] observed the effect of  p27^Kip1^ deletion on CD8 T cell proliferation upon vaccination with recombinant *Listeria monocytogenes. *They found, that initially after vaccination, CD8 T cells exhibited superior recall responses in the absence of p27^Kip1^. Moreover, their study reflects an inhibitory role of p27^Kip1^ in proliferative renewal of memory CD8 T cells, the effector memory subset in particular. The study indicates cell cycle regulation of CD8 T cell homeostasis and supports the idea of modulating p27^Kip1^ to boost vaccine induced T cell memory as well as protective immunity.

Bcl-2, an apoptosis regulatory protein, has also been proposed to be indispensable for the survival of memory cells [47–49]. Several studies relying on double knockout models have proposed a skewing role for Bcl-2. Dunkle et al. [49] performed adoptive transfer experiments, a method to confirm memory potential of cell subsets, exploiting Bcl-2 as a marker which earlier could not be performed owing to the intracellular localization of Bcl-2. They used a novel Bcl-2 reporter mouse model and reported that a distinct subset of effector T cells including a subset within the IL-7*α* ^high^  KLRG1^low^ memory precursor effector cell population retains high Bcl-2 expression at the peak of the CD8 T cell response to *Listeria monocytogenes*. Moreover, their findings enumerate the correlation of Bcl-2 with memory potential in adoptive transfer experiments exploiting both total responding CD8 T cells and memory precursor effector cells. Their results show that Bcl-2, despite being within the memory precursor effector cell population, renders a survival advantage to a subset of effector CD8 T cells allowing their differentiation into memory cells. The findings paint a clear picture of the critical role of Bcl-2 in memory T cell generation. Diacetylated histone H3 (diAcH3) has also been reported as a useful marker for evaluating the functionality of memory CD8 T cells upon *Listeria monocytogenes* infection. Studies performed by DiSpirito and Shen demonstrate that memory T cells impaired in rapid recall response carry less abundant diAcH3 in comparison to their wholesome counterparts which makes diAcH3 level in memory T cells an asset to evaluate their functionality [50].

### 2.8. Kinases As Well Have Some Control over CD8 T Cell Memory Generation

AMPK, adenosine monophosphate-activated protein kinase, gets activated in response to TCR signals upon antigen encounter and during energy stress in T cells as well [51, 52]. It is well documented that AMPK can establish homeostasis and implement dormancy to put down the energy demand in many cell types [53]. Considering these facts, Rolf et al. [53] investigated the role of AMPK during the contraction phase of immune response in regulating the transition of metabolically active effector CD8 T cells to the metabolically dormant catabolic memory T cells. They found that, although AMPK*α*1 activity is not necessarily required for proliferation and differentiation of CTLs, it becomes critical for in vivo survival of CTLs when immune stimulation is withdrawn. They show that T cells lacking AMPK*α*1 were critically defective in their potential to generate memory CD8 T cell responses during *Listeria monocytogenes* infection. Hence, their findings give an insight that AMPK*α*1 controls CD8 T cell memory.

## 3. CD4 T Cells Assist in Memory CD8 T Cell Generation

Factors affecting CD8 T cell response upon bacterial and viral infections are numerous combined with being complex. One of the factors influencing long term survival and fitness of CD8 T memory cell pool is CD4 T cell help [20]. Infection with *Listeria monocytogenes *(either intravenous or enteric) induces clonal expansion of CD4 cells in a manner similar to CD8 T cells [54]. Although assistance of CD4 T cells for the generation of CD8 T cell immune response is overruled during the primary immune response to *Listeria monocytogenes *infection, it plays centrestage for long term maintenance of memory CD8 T cells and establishment of protective immunity against reinfection [20, 55, 56]. Whereas reduced memory in mice lacking CD4 T cell appears only several weeks after infection, CD4 T cells have been found to be critical during the initial priming process, during which they render CD8 T cells with enormous potential to proliferate upon reencounter with the pathogen months later [20, 57]. Hence, CD4 T cell deficient environment renders development and maintenance of CD8 T memory cell pool with reduced magnitude over time affecting the ability of CD8 memory T cells to expand and perform effector functions like cytokine production and hampering their cytotoxic potential which ultimately wanes generation of protective immunity upon rechallenge with the same pathogen. Moreover, it has been demonstrated that memory maintenance stage of CD8 T cell memory requires the presence of CD4 T cells giving insight into the direct or indirect role of CD4 cells to shape the comprehensive quality and functionality of the CD8 memory T cell pool [20, 57, 58].

Though CD4 T cells have been found to impact long term memory CD8 T cells positively, contrastingly, a subset CD4 CD25 regulatory T cells (T_regs_) have been found to suppress *Listeria monocytogenes-*CD8 T cell responses. In CD4 T cell deficient mice, enhanced memory CD8 T cell responses were observed at the time of reinfection in response to immunization, in particular, with peptides or DNA [20, 59]. When CD25 positive cells were depleted, it left a similar effect indicating an inhibitory function of T_regs_ on *Listeria monocytogenes *specific CD8 T cell responses. Interestingly, when wild-type mice are immunized with heat killed *Listeria monocytogenes *(HKLM), protective immunity is not conferred in them, but a higher level of protection is rendered if the mice are depleted in CD4 cells [60]. This suggests that T_regs_ has a restraining effect on CD8 T cell responses following immunization with HKLM, but, on the contrary, T_regs_ are induced less effectively after infection with live, virulent bacteria. 

However, conversely to the above report, a more recent study has unravelled an unexpected function of T_regs_. The findings enumerate that T_regs_ deficit causes activation and expansion of a population of low avidity CD8 cells which leaves the activation of high-avidity T cells impaired during primary immune response resulting in downsized memory to *Listeria monocytogenes. *Considering the results, T_regs_ can be called critical regulators of homeostasis of CD8 T cell priming and inducers of high-avidity primary responses and effective memory [61].

Whereas T cell help is said to be required for the primary response, experiments with noninflammatory immunogens have demonstrated it to be required for generation of secondary response. CD4 T cell help during the initial phase (the first few days) of the immune response has been found to influence the development of a secondary response [57, 62, 63]. As the CD4 T cells are required during the initial phase to program the ensuing CD8 T cell memory response, “unhelped” CD8 T cells, that is, the cells which were not assisted by CD4 during priming, are destined to undergo “activation induced cell death” (AICD) upon restimulation. This is why upon rechallenge ill effects of priming in the absence of CD4 T cell help are seen [30]. Sacks and Bevan [57] have added information with respect to CD4 T cell help. They wondered if TRAIL blockade could rescue impaired CD8 T cell memory in the absence of CD4 help as it has been reported that, in “unhelped” CD8 cells, TRAIL deficit rescues the expansion of restimulated CD8 T cells [30]. They studied memory T cell response in mice doubly deficient in CD4 T cells and TRAIL and found reduced memory CD8 T cell pool in these mice with time and conferred lesser protective immunity upon rechallenge with *Listeria monocytogenes *like their counterparts which were TRAIL sufficient but CD4 unhelped. Hence, Sacks and Bevan conclude* “TRAIL deficiency does not rescue impaired CD8 T cell memory generated in the absence of CD4 T cell help” *[57].

Interestingly, a very recent study highlights that a subunit vaccine that combines polyIC and an agonistic CD40 antibody could “program” protective CD4 independent CD8 T cell memory in response to *Listeria monocytogenes *infection [64].

## 4. B Cells Too Have Some Part to Play in CD8 T Cell Memory Generation

Earlier, the intracellular residence occupied by *Listeria monocytogenes *made the scientific fraternity to conceive the notion that the humoral immune response has to play no role in the control of *Listeria monocytogenes* infection. However, later on, a few findings sprouted in favour of B cell, and antibody influence over *Listeria monocytogenes *infection put that backdrop idea to rest. As B cells have been found to play some part in the generation of T cell response and establishment of memory, they are believed to contribute indirectly in evoking anti-*L. monocytogenes *immunity. Binding of B cell surface and soluble antibodies to their cognate antigens in secondary lymphoid organs, where specific immune responses are initiated, can facilitate the generation of protective T cell response. Whether CD4 and CD8 T cell responses are differentially affected during B cell deficit remains obscure. A study conducted by Shen et al. [65] highlights that, albeit B cells perform a minimal function in the initial activation and antigen-driven expansion of CD8 T lymphocytes, their absence during the contraction phase renders increased death of activated CD8 T cells, leading to reduced *Listeria*-specific CD8 T cell memory. However, B cell influence on long term maintenance and rapid recall response of memory CD8 T cells is denied. Mice deficient in B cells exhibit increased contraction of antigen-specific CD8 T cells, but this possibly is not a consequence of impaired CD4 T cell responses since *Listeria*-specific CD4 T cell priming has been found to be normal in B cell deficient mice [65]. Furthermore, an overcontraction of antigen-specific CD8 T cells was overruled in CD4 deficient mice. Thus, B cells are equipped to perform a specific function of modulating the contraction of CD8 T cell responses following immunization. However, the factors regulating the apoptotic phase remain yet to be unravelled but once elucidated may open avenues to manipulate this process to augment immunological memory and, thus, vaccine efficacy. Furthermore, the old proposition that antigen antibody complex retention on follicular dendritic cells influences the maintenance of long term T cell memory [66] was challenged by later experimental evidence [67–69]. Thus, it becomes very apparent that B cells contribute in modulating T cell responses in *Listeria monocytogenes *and many other similar infections.

## 5. Memory T Cells and Vaccine Development

As discussed earlier, antigen-specific CD4 and CD8 T cells which exist in lower frequencies in naive hosts are the key players in protective immunity. After proliferation in response to antigen, contraction phase begins in which a subpopulation of antigen specific T cells forms memory cell pool. This immunological memory is the basis for vaccination, and wholesome efforts have been put to improve vaccine design through manipulating T cell responses which would maximize memory T cell formation. Although it has been suggested that T_CM_ has a better capacity to replenish memory T cell pool and to mediate protective immunity than T_EM_ because of their greater capacity to proliferate and persist in vivo, the findings of Huster et al. show that T_EM_ is crucial for providing efficient protective immunity against *Listeria monocytogenes* infection [19]. Induction of T_CM_ CD4 cells has also been correlated with prolonged survival, thereby highlighting the importance of gaining a better understanding of the mechanisms underlying central memory induction and persistence for successful vaccine development.

In hosts vaccinated with live *Listeria*-based vaccine, an effective CD8 T cell response is evoked along with generation of sterilizing immunity [70, 71]. On the contrary, the CD8 T cell response to heat killed *Listeria monocytogenes* (HKLM) vaccination does not elicit protective immunity. Interestingly, though HKLM vaccination fails to confer protective immunity, irradiated *Listeria monocytogenes *(IRLM) immunization was successful in protecting mice from secondary infection [71]. However, IRL immunization conferred an inferior protection in comparison to live bacterial infection. Whereas the immunological basis for generation of distinct responses to these three forms of same bacterium remains obscure, Khanna et al. propose that the three forms undergo three different transcriptional and translational programs [71].

Prime-boost vaccination remains the most effective method of choice for inducing higher number of memory T cells combined with increased resistance [14]. However, the classical prime-boost approach requires lengthy time periods between priming and boosting. If the time interval between priming and boosting is shortened, the generated memory cells are downsized to a level below the protective value. Pham et al. using *Listeria monocytogenes *as a model system devised an alternative cross-priming strategy in the absence of adjuvant to shorten the interval between initial priming and booster immunization [29]. It led to the generation of antigen-specific CD8 T cells with augmented memory function which can be amplified with each passing day to achieve protective levels within a very short time. Ansari et al. evaluated the protective efficacy of *Listeria monocytogenes *secretory proteins entrapped in archaeosome (liposome made of Archaea derived lipids) and found that the formulation could generate protective memory in the form of T_CM_ and T_EM_ against *Listeria monocytogenes *infection [2].

## 6. Conclusion

In the last few decades, study of immune response particularly CD8 T cell response to *Listeria monocytogenes* in mouse model has proved to be instrumental for better understanding of intracellular pathogen induced infections. Understanding CD8 T cell memory generation to *Listeria monocytogenes *infection is the focus of numerous research groups across the globe owing to the ray of hope it kindles for designing improved vaccines against a gamut of similar infections. As new cells, receptors, adaptors, and regulatory molecules are discovered each day, *Listeria monocytogenes *is used as a model to decipher their contributions in various host responses including T cell memory generation and till date though it has yielded considerable information on this issue, but the T cell memory picture yet remains petite and requires further studies to get transformed to a comprehensive one.
